# Research hotspots and trends in the relationship between genetics and major depressive disorder: A scientometric analysis from 2003 to 2023

**DOI:** 10.1097/MD.0000000000036460

**Published:** 2023-12-22

**Authors:** Ziwei Zhao, Yanyan Li, Peili Wang, Ran Zhang, Zhongbiao Nie

**Affiliations:** a Affiliated Hospital of Shanxi University of Chinese Medicine, Taiyuan, China; b Shanxi University of Chinese Medicine, Jinzhong, China; c Xiyuan Hospital, China Academy of Chinese Medical Sciences, Beijing, China; d Shanxi Bethune Hospital, Shanxi Academy of Medical Sciences, Tongji Shanxi Hospital, Third Hospital of Shanxi Medical University, Taiyuan, China.

**Keywords:** Citespace, genetics, knowledge mapping, major depressive disorder (MDD), scientometric analysis

## Abstract

To determine current research objectives and predict future trends in studies on the relationship between genetics and major depressive disorder (MDD). We collected the publications in the last 20 years (2003–2023) related to genetics and MDD in the Web of Science database, and applied Citespace to assess the knowledge mapping. The number of manuscripts about genetics and MDD totaled 9200, with a faster increase after 2013. The country, institution, and author with the most publications are the USA, the University of London, and Serretti, Alessandro. BIOL PSYCHIAT published the most articles in this field. In addition, the most co-cited reference is Sullivan PF (2000) (673). Genetic and MDD research, including the hippocampus, and HPA axis may become the focus of research in the future. Based on a 20-year scientometric investigation, we know the USA, China, and Germany have emerged as the important research forces in this discipline. The strongest collaborations between developed countries and renowned institutions are beneficial to the advancement of genetic and MDD research. Serotonin is the strongest citation bursts keyword.

## 1. Introduction

According to the World Health Organization, major depressive disorders (MDD) currently affect 4.4% of the global population.^[1]^ It is anticipated that the COVID-19 pandemic will further raise the prevalence of MDD.^[2]^ Depressive disorders are the single biggest cause of health loss, already costing over 80 million disability-adjusted life years annually, mostly in low- and middle-income nations.^[3]^ Research shows that between 40% and 50% of MDD cases are heritable.^[4]^ Thus, it is believed that there is a significant hereditary component to MDD. Genome-wide association studies (GWASs) have linked variants at numerous loci to MDD.^[4,5]^

Antidepressants’ efficacy in treating MDD varies greatly from patient to patient. Considering these interindividual variations and a variety of antidepressants available, genetic variables can account for a sizable portion of the heterogeneity in responsiveness to antidepressants. In terms of data accessibility and its clinical application, genome sequencing has become incredibly more widely available in recent years.^[6]^ It is essential to grasp the research trends of genetics and MDD. Due to the rapid development of genetic and MDD research, it difficult to fully comprehend its current research state and hotspots. Scientometrics^[7]^ is the quantitative analysis of specific disciplines using a variety of databases including PubMed and Web of Science (WOS)^[8]^ etc. Among them, WOS can conduct scientific quantitative analysis through Citespace software.^[9]^ With Citespace, readers may fully comprehend the hotspots, trends, and frontiers in this sector.^[10,11]^ It explores the key points in the evolution of subject fields. Citespace includes coauthor, co-citation, and co-occurrence analysis.^[12, [13, 14]^

There isn’t a bibliometric method for researching genetics and MDD at the moment. To this end, Citespace was used to analyze the global role and trend of genetics and MDD in the WOS database from January 1, 2003, to March 31, 2023.

## 2. Method

### 2.1. Source of literature

We enter the subject terms into the WOS database: TS = (MDD OR depressive disorder, major OR major depressive disorder OR Severe depression OR depressive disorder OR depressive) AND TS = (gene OR Genetic OR genes OR DNA OR RNA). The search scope of the database is from January 1, 2003, to March 30, 2023, and the language type was English. Through the literature search, 9200 records were obtained. The WOS database comes from the Affiliated Hospital of Shanxi University of Traditional Chinese Medicine database.

### 2.2. Analysis software

Citespace analysis software version is Version 5.6. R2.^[12]^

### 2.3. Download and import of data

The results of the retrieved subject terms were exported, and the file format was kept as “plain text.”

### 2.4. Parameter setting

Time slicing (from 2003 to 2023); node type (checked individually); 50 selection criteria; trimming (pathfinder); and visualization (showing the merged network, cluster view-static).

### 2.5. Statistical methods

**Figure 1.**
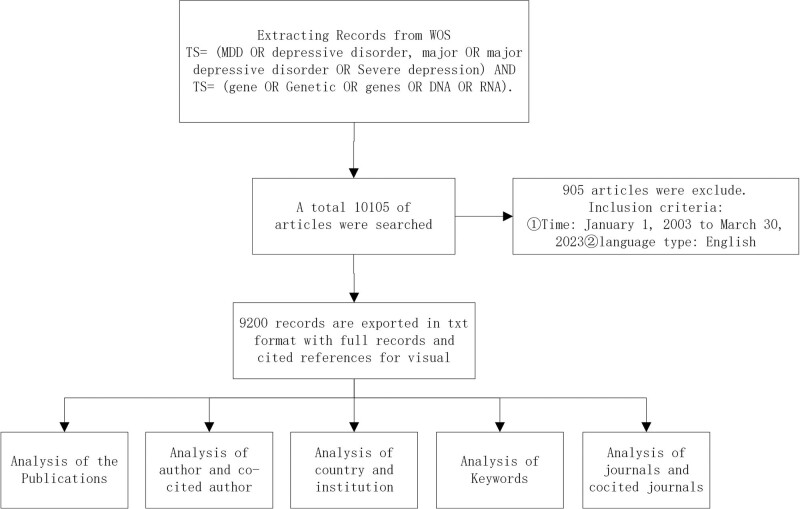
Analysis flow chart of genetics and MDD. MDD = major depressive disorder.

All literature has been scientifically analyzed; Some data obtained include core countries, institutions, authors, keywords, and references.^[13,15,16]^ The detailed analysis flow is shown in Figure 1.

## 3. Results

### 3.1. Analysis of the publications

**Figure 2.**
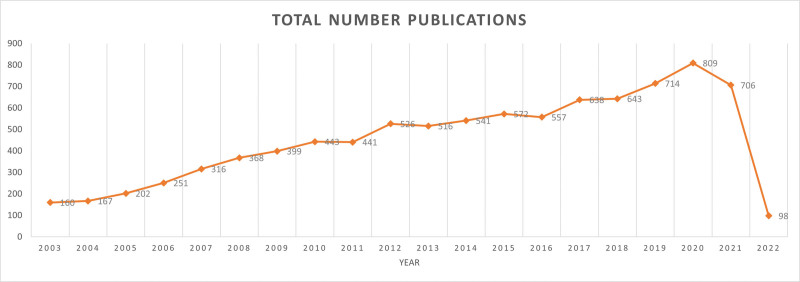
The number of genetic and MDD publications indexed by WOS from 2003 to 2023. MDD = major depressive disorder, WOS = Web of Science.

The overall number of papers increased and fluctuated throughout the research period. As can be seen from Figure 2, the research is divided into 2 stages: the first stage covers 2003 to 2012, and the second stage covers 2013 to 2021. The second stage was a period of rapid development. The publication published 441 references in 2012, rising to 526 references in 2013. In 2021, the number increased to 809. These findings suggest that throughout the previous 8 years, research on genetics and MDD has grown in importance.

### 3.2. Analysis of countries and institutions

**Figure 3.**
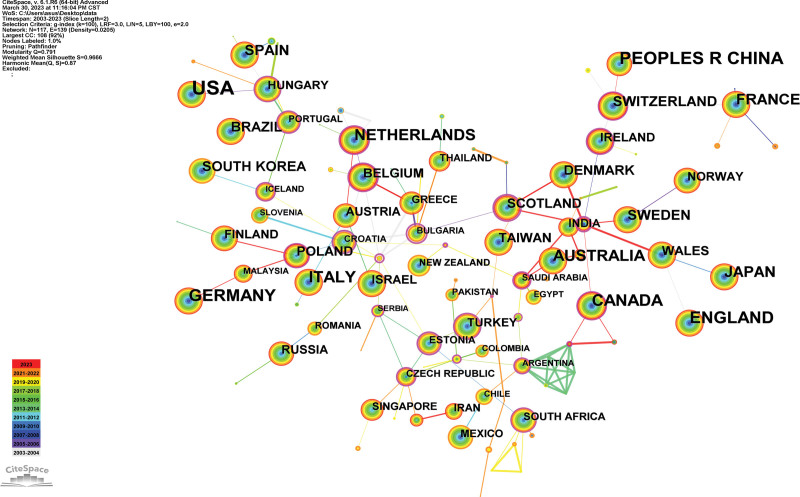
Analysis of the country map from 2003 to 2023.

A country map was generated (Fig. 3). 117 countries published 9057 references. The USA, PEOPLES R CHINA, GERMANY, ENGLAND, and CANADA are the top 5 countries (Table 1). The USA (0.18), and FRANCE (0.15) are the top 2 countries from centrality (purple round). An analysis of publications and centrality shows that the USA, PEOPLES R CHINA, and GERMANY were the main research forces in the study of the relationship between genetics and MDD. FRANCE, ENGLAND, and CANADA have been increasingly interested in this field. The research was distributed mainly in developed countries, and cooperation between countries was weak.

**Table 1:** Top 5 countries and institutions researching between genetic and MDD.

Ranking	Country	Publications	Ranking	Institution	Publications
1	USA	3522	1	University of London	501
2	Peoples R China	1206	2	University of California System	496
3	Germany	970	3	Harvard University	433
4	England	933	4	King College London	404
5	Canada	792	5	National Institutes of Health (NIH)—USA	311

MDD = major depressive disorder.

**Figure 4.**
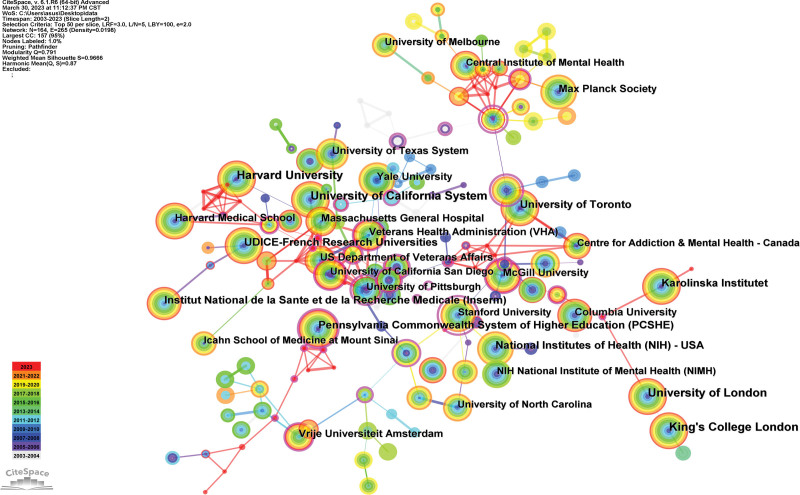
Institutional map researching genetic and MDD from 2003 to 2023. MDD = major depressive disorder.

Generated an institution map with 164 nodes and 265 links (Fig. 4). The 9081 publications have been published in 164 institutions. The University of London, the University of California System, Harvard University, King College London, and the National Institutes of Health are the top 5 institutions (Table 1). Regarding centrality, the top 3 institutions were Harvard University (0.16), Pennsylvania Commonwealth System of Higher Education (0.14), and the University of Bonn (0.13). In addition, the relatively thin links between various institutions indicate weak cooperation.

### 3.3. Analysis of journals

**Figure 5.**
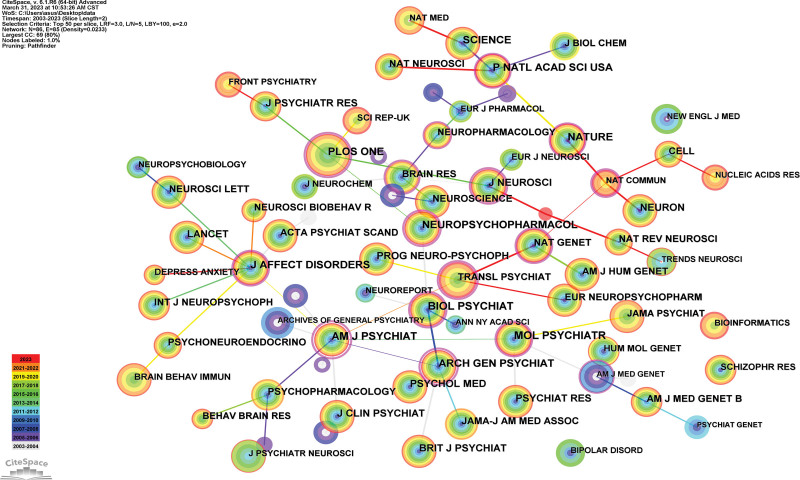
Journal map researching genetic and MDD from 2003 to 2023. MDD = major depressive disorder.

The top 5 journals between genetics and MDD (Table 2). They are professional journals in this field. Generated a journal map that had 86 nodes and 85 links (Fig. 5). BIOL PSYCHIAT, MOL PSYCHIATR, AM J PSYCHIAT, ARCH GEN PSYCHIAT, J AFFECT DISORDERS are the top 5 journals.

**Table 2:** Top 5 academic journals related to research between genetic and MDD.

Ranking	Journal	Publications	IF (2021)
1	Biol Psychiat	5958	12.810
2	Mol Psychiatr	5539	13.437
3	Am J Psychiat	5207	19.242
4	Arch Gen Psychiat	4694	-
5	J Affect Disorders	4514	6.533

MDD = major depressive disorder.

### 3.4. Analysis of author

**Figure 6.**
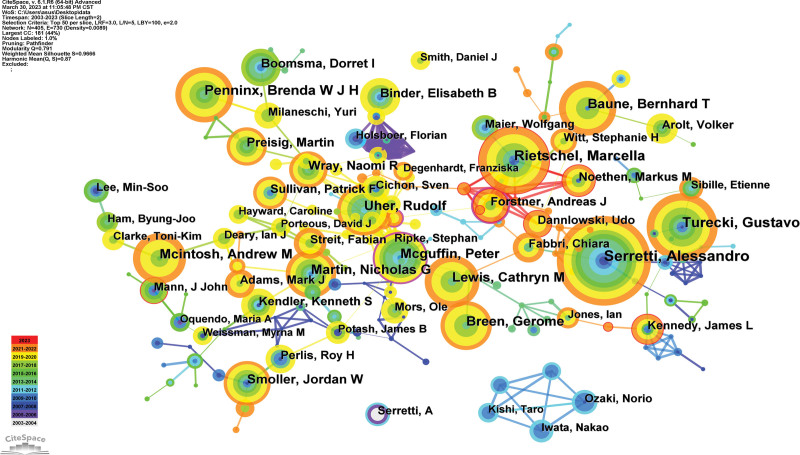
Author map researching genetic and MDD from 2003 to 2023. MDD = major depressive disorder.

405 authors published 8772 articles. The top 5 authors who have been written about (Table 3) are experts in this field. The generated map had 405 nodes and 730 links (Fig. 6).

**Table 3:** Top 5 Authors in between genetic and MDD research from 2003 to 2023.

Ranking	Author	Publications
1	Serretti, Alessandro	110
2	Rietschel, Marcella	84
3	Turecki, Gustavo	83
4	Penninx, Brenda W J H	68
5	Baune, Bernhard T	67

MDD = major depressive disorder.

As the author with the most published articles, Alessandro Serretti is based at the Department of Biomedical and Neuromotor Sciences. Their team has made a detailed study on the genetic epidemiology of major depression, to investigate the likelihood of these disorders having a common genetic basis, merging some of the top scientific tools in genetics. The combination of qualitative and quantitative studies of the shared genetic characteristics linked to sensitivity to these illnesses is the primary novel contribution to the current study.^[17]^

### 3.5. Analysis of co-cited references

An analysis of counts and centrality (see Tables S1, S2. Supplemental Table S1, http://links.lww.com/MD/L46, http://links.lww.com/MD/L47, which demonstrates top 5 co-cited references in terms of co-citation counts. Supplemental Table S2, http://links.lww.com/MD/L47, which demonstrates top 5 co-cited references in terms of centrality. See Fig. S1, Supplemental Fig. S1, http://links.lww.com/MD/L44, which demonstrates co-citation map of reference) revealed that the data usually comes in the form of a review. Among them, “Genetic Epidemiology of major depression: review and Meta-analysis” was published in Am J Psychiatry in 2000. Sullivan PF experimentally confirmed that major depression is a familial condition, and genetic factors are mostly or fully responsible for its prevalence. Also, an individual unique environmental factors have etiological significance. The complex condition known as major depression is caused by both hereditary and environmental factors, rather than just one or the other.^[4]^ They have had a significant influence on this field of research. Two genetic alterations or variations have been linked to MDD in studies, one in the SIRT1 gene and the other in an intron (a non-protein-coding area) of the LHPP gene.^[18,19]^ His work has made significant contributions to genetics and MDD.

### 3.6. Analysis of keywords and burst keywords

Figure S2 (Supplemental Fig. S2, http://links.lww.com/MD/L45, which demonstrates the co-occurrence network of keywords map) shows the occurrence network of keywords. The analysis divided them into 10 clusters. In cluster 1, the high-rate keywords were “stress (186.82, 1.0E-4), hippocampus (169.59, 1.0E-4), HPA axis (116.37, 1.0E-4).” In cluster 2, the high-rate keywords were “major depressive disorder (343.54, 1.0E-4), and pharmacogenetics (219.67, 1.0E-4).” In cluster 3, the high-rate keywords were “mood disorders (124.73, 1.0E-4), suicide (121.71, 1.0E-4),” and in cluster 4, “depressive disorder (76.09, 1.0E-4) and mendelian randomization.” Keywords can reflect the hotspots and research directions in the field of research to a certain extent. The analysis of keywords indicates that the hippocampus and HPA axis may become the focus of research in the future.

**Figure 7.**
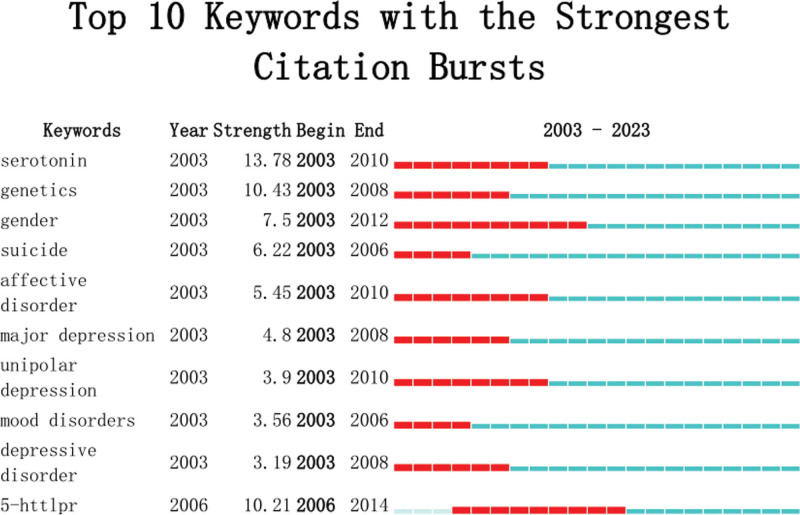
Top 10 keywords with the strongest citation bursts.

Burst words are considered indicators for frontier topics over time and are frequently cited over some time (Fig. 7). Three frontiers in genetics and MDD were serotonin, genetics, and gender.

## 4. Discussion

From the analysis of Citespace, we found that the annual articles about genetics and MDD increased in number from 2003 to 2023. It may be because genetics is an important component of MDD, and more and more researchers attach importance to it.

The amount of gene studies on MDD is growing, and scientists are paying more attention to the genetic pharmacological effects of antidepressants. Two significant SNPs (rs6966038, 51 kb from UBE3C and 77 kb from MNX1) were related to citalopram responsiveness in one STAR*D GWAS that was focused on medication response and remission rate.^[20]^ Another study using the same data discovered that IL28RA and PAPLN variations are linked to suicidal ideation, a side effect of citalopram therapy.^[21]^ SNPs in the NRXN3 and ITGA9 (integrin, alpha 9 subunit gene) were discovered by a combined GENDEP and STAR*D GWAS study (neurexin-3-alpha gene) that were associated with improved symptoms during citalopram/escitalopram treatment. The ITGA9 SNP, however, was the only one of these correlations which have been repeated in one of the 2 additional datasets used for validation.^[22]^ There is strong evidence that proper application of the pharmacogenetic tests already in use may increase remission rates. However, there are differences between the tests, necessitating the development of more precise DSTs. Pharmacogenetic testing may have already used next-generation sequencing data today, for instance, targeted sequencing which converts sequencing data of known pharmacogenetics into STAR genotypes.^[23]^

We note that the hippocampus may become the focus of research in the future. The hippocampus is a crucial limbic area that is predominantly linked to memory.^[24,25]^ Growing data supports the hippocampus function in MDD. Ingo J. Numerous studies have shown that MDD also caused a decrease in the volume of the granule cell layer and the number of hippocampal granule cells.^[26,27]^ Mahajan^[28]^ identifies the presence and type of gene expression variations linked to MDD in the hippocampus.

Serotonin is the strongest citation burst keywords, representing a research hotspot and trend of antidepressant drugs. The increased clearance of the neurotransmitter serotonin from the synaptic cleft, also known as 5-hydroxytryptamine, is thought to be one of the biochemical causes of MDD. As a result, a lot of studies have concentrated on searching for polymorphisms in serotonin transporter genes.^[29]^

We found that after 2012, the number of published articles in this field increased significantly, indicating that the research heat in this field increased significantly, probably in 2012, Kupfer et al^[30]^ published “Major depressive disorder: new clinical, neurobiological, and treatment perspectives” in the Lancet. It indicated that genetic studies continue to contribute to advances in our understanding of the neurobiological basis of MDD. GWASs further suggest that effectiveness of antidepressants can be predicted by genetic markers other than traditional candidate genes.^[31]^

In the past 20 years, genetic research of depression has become 2-tiered to: understand the pathophysiology of the illness; identify the neurobiological measures for guiding treatment choice; and effectiveness of antidepressants can be predicted by genetic markers other than traditional candidate genes.^[32]^

Clinical studies have shown that a deficit in serotonin (5-HT) is a putative biomarker for MDD. Indeed, clinical studies found reduced cerebrospinal fluid and plasma concentrations of the 5-HT major metabolite—5-hydroxyindoleacetic acid—in drug-free depressed patients that was associated with higher suicidal attempts,^[33,34]^ suggesting an altered 5-HT turnover rate in MDD. Consistent with this evidence, treatment with para-chloro-phenylalanine, a tryptophan hydroxylase inhibitor, that depleted central 5-HT system, caused a rapid relapse in depressed patients who had responded to the antidepressant drug medication.^[35]^ Therefore, drugs targeting the serotonin transporter—namely, SSRIs and SNRIs—have been used for the treatment of MDD.

## 5. Conclusion

Genetic Variations are undoubtedly associated with mental disease and account for a large proportion of the inter-individual variation in pharmacotherapeutic response (pharmacogenetics). Of all the medical practices, psychiatry most clearly stands to represent the unique genetic and epigenetic interaction of gene expression in an individual patient. Bibliometric analysis of genetic and MDD publications from 2003 to 2023 revealed that serotonin, genetics and gender may be the focus of future research. With strong publishing rates and centrality, the USA, People Republic of China, and Germany have emerged as the 3 major research nations in this area. The strongest collaborations between developed nations and renowned institutions are beneficial to the advancement of genetic and MDD research. Serotonin was the strongest citation bursts keywords. These articles were widely cited because they are a guideline or have a high IF.

## Acknowledgments

The authors would like to express their appreciation to Professor Chaomei Chen for inventing Citespace and making it free to use.

## Author contributions

**Conceptualization:** Ziwei Zhao, Ran Zhang, Zhongbiao Nie.

**Data curation:** Ziwei Zhao, Ran Zhang, Zhongbiao Nie.

**Funding acquisition:** Yanyan Li.

**Investigation:** Ziwei Zhao, Yanyan Li.

**Methodology:** Yanyan Li, Peili Wang, Zhongbiao Nie.

**Project administration:** Peili Wang, Zhongbiao Nie.

**Resources:** Zhongbiao Nie.

**Writing – original draft:** Ziwei Zhao, Zhongbiao Nie.

**Writing – review & editing:** Zhongbiao Nie.

## Supplementary Material








