# Supplementary material for: Research hotspots and trends in the relationship between genetics and major depressive disorder: A scientometric analysis from 2003 to 2023
Source: Medicine (Baltimore). 2023 Dec 22;102(51):e36460. doi: 10.1097/MD.0000000000036460 (PMC10735073; doi:10.1097/MD.0000000000036460)
Supplement: Supplementary file 1 [file medi-102-e36460-s001.docx]

**Table S1** Top 5 co-cited references related to genetic and MDD research in terms of co-citation

| **Ranking** | **Cited reference** | **Co-citation counts** | **Representative author (publication year)** |
| --- | --- | --- | --- |
| **1** | Genetic epidemiology of major depression: review and meta-analysis^[1]^ | 673 | Sullivan P F (2000) |
| **2** | influence of Life Stress on Depression: Moderation by a Polymorphism in the 5-HTT Gene^[2]^ | 656 | Caspi A (2003) |
| **3** | A rating scale for depression^[3]^ | 492 | HAMILTON M (1960) |
| **4** | PLINK: a tool set for whole-genome association and population-based linkage analyses^[4]^ | 376 | Purcell S (2007) |
| **5** | The epidemiology of major depressive disorder: results from the National Comorbidity Survey Replication (NCS-R)^[5]^ | 354 | Kessler RC (2003) |

**参考文献**

[1] Sullivan P F, Neale M C, Kendler K S. Genetic epidemiology of major depression: review and meta-analysis[J]. Am J Psychiatry, 2000,157(10):1552-1562.

[2] Caspi A, Sugden K, Moffitt T E, et al. Influence of life stress on depression: moderation by a polymorphism in the 5-HTT gene[J]. Science, 2003,301(5631):386-389.

[3] HAMILTON M. A rating scale for depression[J]. J Neurol Neurosurg Psychiatry, 1960,23(1):56-62.

[4] Purcell S, Neale B, Todd-Brown K, et al. PLINK: a tool set for whole-genome association and population-based linkage analyses[J]. Am J Hum Genet, 2007,81(3):559-575.

[5] Kessler R C, Berglund P, Demler O, et al. The epidemiology of major depressive disorder: results from the National Comorbidity Survey Replication (NCS-R)[J]. JAMA, 2003,289(23):3095-3105.

**校对报告**

当前使用的样式是 [中华人民共和国国家标准_GBT_7714-2005]

当前文档题录总数为5条，在5个位置共计插入5次（包括重复插入）

有0条题录存在必填字段内容缺失的问题

所有题录的数据正常
