# Supplementary material for: Research hotspots and trends in the relationship between genetics and major depressive disorder: A scientometric analysis from 2003 to 2023
Source: Medicine (Baltimore). 2023 Dec 22;102(51):e36460. doi: 10.1097/MD.0000000000036460 (PMC10735073; doi:10.1097/MD.0000000000036460)
Supplement: Supplementary file 2 [file medi-102-e36460-s002.docx]

**Table S2** Top five co-cited references in between genetic and MDD research in terms of centrality

| **Ranking** | **Cited reference** | **centrality** | **Representative author (publication year)** |
| --- | --- | --- | --- |
| 1 | Genetic epidemiology of major depression: review and meta-analysis^[1]^ | 0.44 | Sullivan P F (2000) |
| 2 | influence of Life Stress on Depression: Moderation by a Polymorphism in the 5-HTT Gene^[2]^ | 0.25 | Caspi A (2003) |
| 3 | The BDNF val66met polymorphism affects activity-dependent secretion of BDNF and human memory and hippocampal function^[3]^ | 0.18 | Egan MF (2003) |
| 4 | Chapter 6-The Neurotrophic Hypothesis of Depression Revisited: New Insights and Therapeutic Implications^[4]^ | 0.1 | Duman RS (2006) |
| 5 | Common polygenic variation contributes to risk of schizophrenia and bipolar disorder^[5]^ | 0.1 | Purcell SM (2009) |

**参考文献**

[1] Sullivan P F, Neale M C, Kendler K S. Genetic epidemiology of major depression: review and meta-analysis[J]. Am J Psychiatry, 2000,157(10):1552-1562.

[2] Caspi A, Sugden K, Moffitt T E, et al. Influence of life stress on depression: moderation by a polymorphism in the 5-HTT gene[J]. Science, 2003,301(5631):386-389.

[3] Egan M F, Kojima M, Callicott J H, et al. The BDNF val66met polymorphism affects activity-dependent secretion of BDNF and human memory and hippocampal function[J]. Cell, 2003,112(2):257-269.

[4] Jaggar M, Fanibunda S E, Ghosh S, et al. Chapter 6 - The Neurotrophic Hypothesis of Depression Revisited: New Insights and Therapeutic Implications[J]. Neurobiology of Depression, 2019:43-62.

[5] Purcell S M, Wray N R, Stone J L, et al. Common polygenic variation contributes to risk of schizophrenia and bipolar disorder[J]. Nature, 2009,460(7256):748-752.

**校对报告**

当前使用的样式是 [中华人民共和国国家标准_GBT_7714-2005]

当前文档题录总数为5条，在5个位置共计插入5次（包括重复插入）

有0条题录存在必填字段内容缺失的问题

所有题录的数据正常
