# Supplementary figures and images for: Research hotspots and trends in the relationship between genetics and major depressive disorder: A scientometric analysis from 2003 to 2023
Source: Medicine (Baltimore). 2023 Dec 22;102(51):e36460. doi: 10.1097/MD.0000000000036460 (PMC10735073; doi:10.1097/MD.0000000000036460)

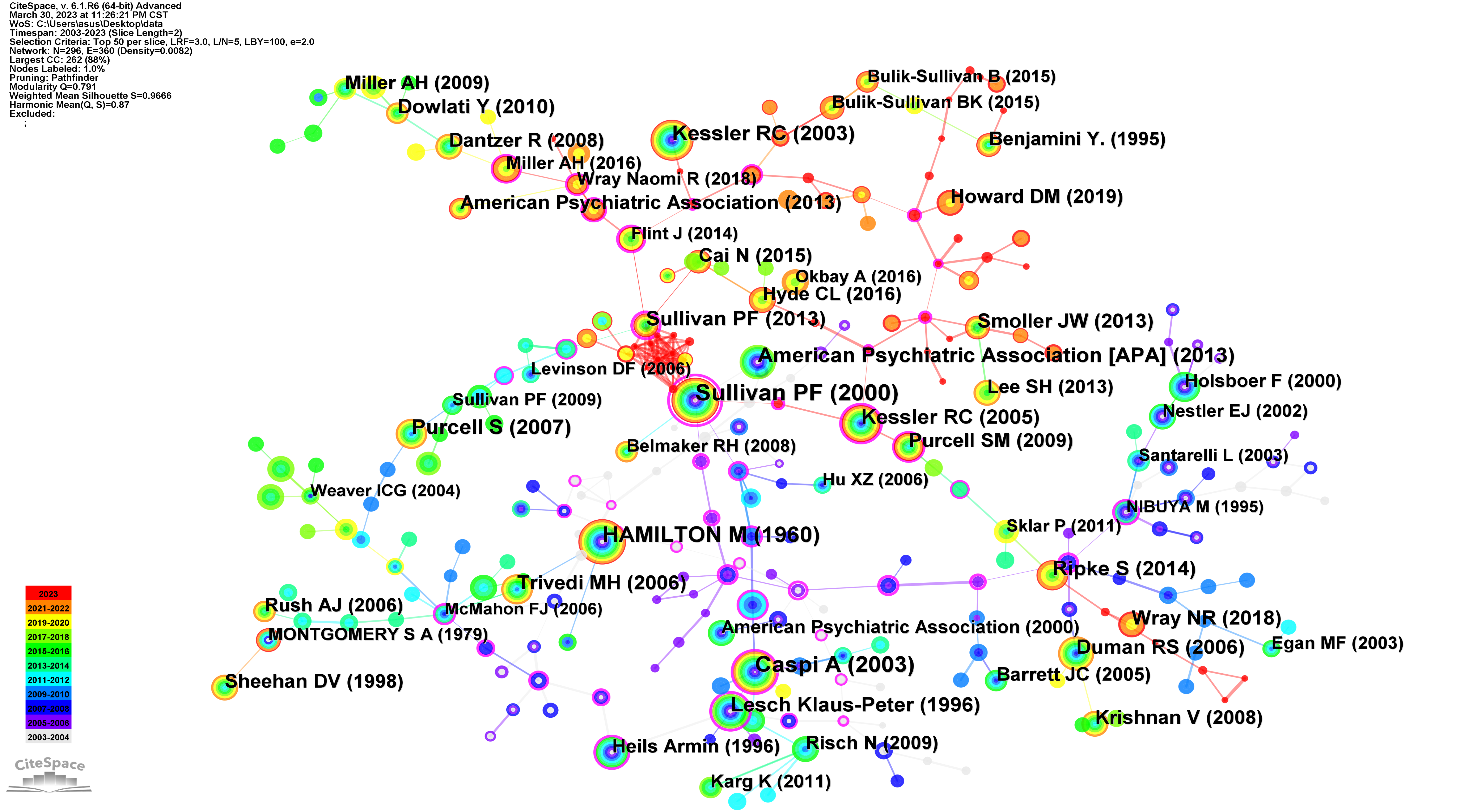

Supplement: Supplementary file 3 [file medi-102-e36460-s003.tiff]

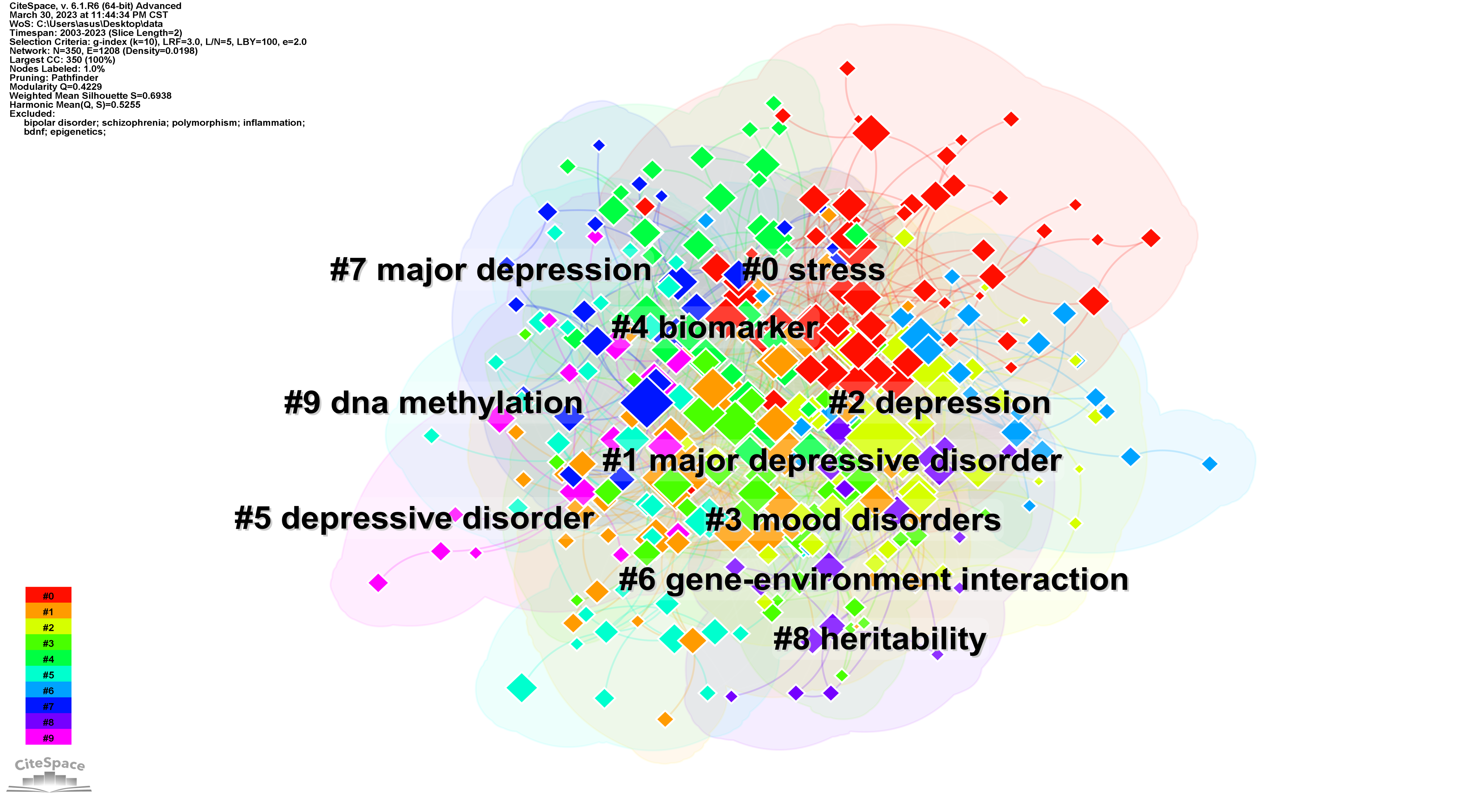

Supplement: Supplementary file 4 [file medi-102-e36460-s004.tiff]
